# Entropic Uncertainty Relations for Successive Measurements in the Presence of a Minimal Length

**DOI:** 10.3390/e20050354

**Published:** 2018-05-09

**Authors:** Alexey E. Rastegin

**Affiliations:** Department of Theoretical Physics, Irkutsk State University, Gagarin Bv. 20, 664003 Irkutsk, Russia; alexrastegin@mail.ru

**Keywords:** generalized uncertainty principle, successive measurements, minimal observable length, Rényi entropy, Tsallis entropy

## Abstract

We address the generalized uncertainty principle in scenarios of successive measurements. Uncertainties are characterized by means of generalized entropies of both the Rényi and Tsallis types. Here, specific features of measurements of observables with continuous spectra should be taken into account. First, we formulated uncertainty relations in terms of Shannon entropies. Since such relations involve a state-dependent correction term, they generally differ from preparation uncertainty relations. This difference is revealed when the position is measured by the first. In contrast, state-independent uncertainty relations in terms of Rényi and Tsallis entropies are obtained with the same lower bounds as in the preparation scenario. These bounds are explicitly dependent on the acceptance function of apparatuses in momentum measurements. Entropic uncertainty relations with binning are discussed as well.

## 1. Introduction

The Heisenberg uncertainty principle [1] is now avowed as a fundamental scientific concept. Heisenberg examined his thought experiment rather qualitatively. An explicit formal derivation appeared in [2]. This approach was later extended to arbitrary pairs of observables [3]. These traditional formulations are treated as preparation uncertainty relations [4], since repeated trials with the same quantum state are assumed here. This simple scenario differs from the situations typical in quantum information science. Since uncertainty relations are now examined not only conceptually, researchers often formulated them in information-theoretic terms. As was shown in [5], wave-particle duality can be interpreted on the basis of entropic uncertainty relations. Basic developments within the entropic approach to quantum uncertainty are reviewed in [6,7,8]. Interest in this approach has been stimulated by advances in using quantum systems as an informational resource [9,10,11,12,13]. Among more realistic cases, scenarios with successive measurements have been addressed in the literature [14,15,16,17,18]. Researchers are currently able to manipulate individual quantum systems [19,20]. In quantum information processing, our subsequent manipulations usually deal with an output of a latter stage. In effect, Heisenberg’s thought experiment with microscope should rather be interpreted as related to uncertainties in successive measurements [21]. Uncertainty relations in the scenarios of successive measurements have received less attention than they deserve [15]. The authors of [15] also compared their findings with noise-disturbance relations given in [22]. Studies of scenarios with successive measurements allow us to understand whether preparation uncertainty relations are applicable to one or another question.

In principle, the Heisenberg uncertainty principle does not impose a restriction separately on spreads of position and momentum. It merely reveals that continuous trajectories are unspeakable in standard quantum mechanics, although such principles remain valid within Bohmian mechanics [23]. The generalized uncertainty principle is aimed to involve the existence of a minimal observable length. The latter is naturally connected with efforts to describe quantum gravity [24]. Some advances in merging quantum mechanics and general relativity are summarized in [25]. It is believed that quantum gravitational effects begin to be apparent at the scale corresponding to the Planck length $\ell_{P}=\sqrt{G\hbar /c^{3}}\approx 1.616\times 10^{-35}$ m. Below this scale, the very structure of space-time is an open problem [26]. In addition, Heisenberg’s principle is assumed to be converted into the generalized uncertainty principle (GUP) [27,28,29]. There exist proposals to test observable effects of the minimal length, including astronomical observations [30,31] and experimental schemes feasible within current technology [32,33]. The GUP case connects to many aspects that are currently the subject of active research [34,35,36,37,38]. The generalized uncertainty principle declares a non-zero lower bound on position spread. To reach such a model, the canonical commutation relation should be modified. Deformed forms of the commutation relation were recently studied from several viewpoints. On the other hand, the connections of the GUP with the real world represent an open question. In the context of non-relativistic quantum mechanics, the corresponding formalism was proposed in [39]. Another approach to representation of the used observables was suggested in [40]. This way is very convenient in extending entropic uncertainty relations to the GUP case [41].

In this paper, we aim to consider entropic uncertainty relations for successive measurements in the presence of a minimal observable length. Of course, our presentation is essentially based on mathematical relations given by Beckner [42] and by Białynicki-Birula and Mycielski [43]. This direction was initially inspired by Hirschman [44]. For observables with finite spectra, basic developments appeared due to [45,46,47]. We will largely use the results reported in [48,49]. The work in [48] is devoted to formulating entropic uncertainty relations for successive measurements of canonically conjugate observables. The case of position and momentum was addressed therein as a particular example of the scheme developed in [50,51]. Entropic uncertainty relations in the presence of a minimal length were examined in [49], and mainly focused on those points that were not considered in this context previously. Combining these two aspects finally led to the generalized uncertainty principle in scenarios of successive measurements. This paper is organized as follows. In Section 2, we review preliminary material, including properties of used information-theoretic measures. In Section 3, we briefly discuss successive quantum measurements in general. The main results of this paper are presented in Section 4. Both of the typical scenarios of successive measurements will be examined. In particular, we will see how formulating lower entropic bounds depends on the actual order in which measurements of position and momentum have been performed. In Section 5, we conclude the paper with a summary of the obtained results.

## 2. Preliminaries

In this section, we review the required material and fix the notations. To characterize measurement uncertainties, we use entropies of the Rényi and Tsallis types. Let us begin with the case of probability distributions with a discrete label. For the given probability distribution $p=\{p_{i}\}$, its Rényi entropy of order α is defined as [52]

(1) $$R_{\alpha }\left(p\right):=\frac{1}{1-\alpha }\;\ln\left(\sum_{i}p_{i}^{\alpha }\right),$$

where 0<α≠1. For 0<α<1, the Rényi α-entropy is a concave function of the probability distribution. For α>1, it is neither purely convex nor purely concave [53]. In the limit α→1, the formula (1) gives the standard Shannon entropy

(2) $$H_{1}\left(p\right)=-\sum_{i}p_{i}\ln p_{i}\;.$$

For the given probability distribution $p=\{p_{i}\}$ and 0<α≠1, the Tsallis α-entropy is defined as [54]

(3) $$H_{\alpha }\left(p\right):=\frac{1}{1-\alpha }\;\left(\sum_{i}p_{i}^{\alpha }-1\right)=-\sum_{i}p_{i}^{\alpha }\ln_{\alpha }\left(p_{i}\right).$$

Here, we use the α-logarithm expressed as $\ln_{\alpha }\left(y\right):=\left(y^{1-\alpha }-1\right)/\left(1-\alpha \right)$ for positive variable *y* and 0<α≠1. When α→1, the α-logarithm reduces to the usual one. Then, the α-entropy (3) also leads to the Shannon entropy (2). An axiomatic approach to generalized information-theoretic quantities is reviewed in [55]. In more detail, properties and applications of generalized entropies in physics are discussed in [56]. In the present paper, we will deal only with entropies of probability distributions. Quantum entropies of very general family were thoroughly examined in [57,58]. Quantum Rényi and Tsallis entropies are both particular representatives of this family.

Let w(x) be a probability density function defined for all real *x*. Then, the differential Shannon entropy is introduced as

(4) $$H_{1}\left(w\right):=-\int_{-\infty }^{+\infty }w\left(x\right)\;\ln w\left(x\right)\;dx\;.$$

Similarly, we determine entropies for other continuous variables of interest. For 0<α≠1, the differential Rényi α-entropy is defined as

(5) $$R_{\alpha }\left(w\right):=\frac{1}{1-\alpha }\;\ln\;\left(\int_{-\infty }^{+\infty }w{\left(x\right)}^{\alpha }\;dx\right).$$

In contrast to entropies of a discrete probability distribution, differential entropies are not positive definite in general. To quantify an amount of uncertainty, we often tend to deal with positive entropic functions. One possible approach is such that the continuous axis of interest is divided into a set of non-intersecting bins. Preparation uncertainty relations with binning were derived in terms of the Shannon [59] and Rényi entropies [60]. To reach a good exposition, the size of these bins should be sufficiently small in comparison with a scale of considerable changes of w(x). Keeping an obtained discrete distribution, we further calculate entropies of the forms (1) and (3).

The generalized uncertainty principle declares the deformed commutation relation for the position and momentum operators [39]. For convenience, we will use the wavenumber operator $\hat{k}$ instead of the momentum operator $\hbar \hat{k}$. It is helpful to rewrite this relation as

(6) $$\left[\hat{x},\hat{k}\right]=i\left(1+\beta {\hat{k}}^{2}\right)\;.$$

Here, the positive parameter β is assumed to be rescaled by factor $\hbar^{2}$ from its usual sense. With the limit β→0, the formula (6) gives the standard commutation relation of ordinary quantum mechanics. Due to the Robertson formulation [3], the standard deviations in the pre-measurement state $\hat{\rho }$ satisfy

(7) $$\Delta \hat{A}\;\Delta \hat{B}\geq \left|\frac{1}{2}\;{\langle [\hat{A},\hat{B}]\rangle }_{\hat{\rho }}\right|\;.$$

By ${\langle \hat{A}\rangle }_{\hat{\rho }}=\mathrm{Tr}\left(\hat{A}\;\hat{\rho }\right)$, we mean the quantum-mechanical expectation value. Combining (6) with (7) then gives

(8) $$\Delta \hat{x}\;\Delta \hat{k}\geq \frac{1}{2}\;\left(1+\beta {\langle {\hat{k}}^{2}\rangle }_{\hat{\rho }}\right)\geq \frac{1}{2}\;\left(1+\beta {\left(\Delta \hat{k}\right)}^{2}\right)\;.$$

The principal parameter β is positive and independent of $\Delta \hat{x}$ and $\Delta \hat{k}$ [39]. It directly follows from (8) that $\Delta \hat{x}$ is not less than the square root of β. As was shown in [40], the auxiliary wavenumber operator $\hat{q}$ allows us to mediate between (6) and the standard commutation relation. Let $\hat{x}$ and $\hat{q}$ be self-adjoint operators satisfying $[\hat{x},\hat{q}]=i$. In the *q*-space, the action of $\hat{q}$ results in multiplying a wave function φ(q) by *q*, whereas $\hat{x}\varphi \left(q\right)=i\;d\varphi /dq$. Then, the wavenumber $\hat{k}$ can be represented as [40]

(9) $$\hat{k}=\frac{1}{\sqrt{\beta }}\;\tan\left(\sqrt{\beta }\hat{q}\right)\;.$$

The auxiliary wavenumber obeys the standard commutation relation but ranges between $\pm \;q_{0}\left(\beta \right)=\pm \;\pi /\left(2\sqrt{\beta }\;\right)$. The function $q\mapsto k=\tan\left(\sqrt{\beta }q\right)/\sqrt{\beta }$ gives a one-to-one correspondence between $q\in (-\;q_{0};+\;q_{0})$ and k∈(−∞;+∞). Hence, the eigenvalues of $\hat{k}$ fully cover the real axis. Further details of the above representation are examined in [40].

For any pure state, we will deal with three wave functions ϕ(k), φ(q), and ψ(x). The formalism of [40] is convenient in the sense that it explicitly describes the space of acceptable wave packets. In the *q*-space, these states should have wave functions that vanish for $|q|>q_{0}\left(\beta \right)$. Here, the auxiliary wave function φ(q) is a useful tool related to ψ(x) via the Fourier transform. In the *q*-space, the eigenfunctions of $\hat{x}$ appear as $\exp\left(-iqx\right)/\sqrt{2\pi }$. Thus, any wave function in the coordinate space is expressed as

(10) $$\psi \left(x\right)=\frac{1}{\sqrt{2\pi }}\int_{-q_{0}}^{+q_{0}}\exp\left(+iqx\right)\;\varphi \left(q\right)\;dq\;.$$

Wave functions in the *q*- and *x*-spaces are connected by the Fourier transform [40],

(11) $$\varphi \left(q\right)=\frac{1}{\sqrt{2\pi }}\int_{-\infty }^{+\infty }\exp\left(-iqx\right)\;\psi \left(x\right)\;dx\;.$$

The distinction from ordinary quantum mechanics is that wave functions in the *q*-space should be formally treated as 0 for all $|q|>q_{0}\left(\beta \right)$.

Using the above connection, the author of [41] affirmed the following. The uncertainty relation given in [42,43] is still valid in the GUP case. However, wave functions in the *q*-space are actually auxiliary. In the GUP case, the physically legitimate wavenumber and momentum involved in the relation (6) are described by wavefunctions in the *k*-space. A real distribution of physical wavenumber values is determined with respect to ϕ(k) instead of φ(q). Let us examine the probability that momentum lies between two prescribed values. In view of the bijection between the intervals $\left(k_{1};k_{2}\right)$ and $\left(q_{1};q_{2}\right)$, this probability is expressed as

(12) $$\int_{k_{1}}^{k_{2}}{\left|\phi \left(k\right)\right|}^{2}\;dk=\int_{q_{1}}^{q_{2}}{\left|\varphi \left(q\right)\right|}^{2}\;dq\;,$$

so that ${\left|\phi \left(k\right)\right|}^{2}\;dk={\left|\varphi \left(q\right)\right|}^{2}\;dq$. Hence, two probability density functions u(k) and v(q) are connected as u(k)dk=v(q)dq, in another form

(13) $$u\left(k\right)=\frac{v(q)}{1+\beta k^{2}}\;.$$

For pure states, when $u\left(k\right)={\left|\phi \left(k\right)\right|}^{2}$ and $v\left(q\right)={\left|\varphi \left(q\right)\right|}^{2}$, the formula (13) is obvious. It can be extended to mixed states due to the spectral decomposition. However, one is actually unable to obtain the probability density functions u(k) and w(x) immediately.

In reality, any measurement apparatus is inevitably of a finite size. Devices with a finite extension need a finite amount of energy. Hence, one cannot ask for a state in which the measurement of an observable gives exactly one particular value of position. In more detail, measurements of coordinates of a microparticle are considered by Blokhintsev ([61], Chapter II). The generalized uncertainty principle imposes another limitation for position measurements. Although eigenstates of position and momentum are often considered explicitly, they are rather convenient tools of mathematical technique. The corresponding kets are not elements of the Hilbert space, but can be treated in the context of rigged Hilbert spaces [62]. Instead, we aim to use narrow distributions of a finite but small width. Measuring or preparing some state with the particular value ξ of position, one has to be affected by a neighborhood of ξ. Therefore, we treat each concrete result only as an estimation compatible with the GUP.

Thus, we cannot directly obtain probability density functions of the form u(k) and w(x). Here, a finiteness of detector resolution should be addressed [15,48]. Measuring or preparing a state with the particular value ξ of position, one is affected by some vicinity of ξ. In this way, we refer to generalized quantum measurements. Let the eigenkets |x〉 be normalized through Dirac’s delta function. As was already mentioned, such kets cannot be treated as physical states even within ordinary quantum mechanics. In a finite-resolution measurement of position, the set $X=\left\{|x\rangle \langle x|\right\}$ is replaced with some set N of operators of the form

(14) $$\hat{N}\left(\xi \right):=\int_{-\infty }^{+\infty }dx\;g\left(\xi -x\right)\;|x\rangle \langle x|\;.$$

An acceptance function ξ↦g(ξ) satisfies the condition $\int_{-\infty }^{+\infty }{\left|g\left(\xi \right)\right|}^{2}\;d\xi =1$. Then operators of the form (14) lead to a generalized resolution of the identity,

(15) $$\int_{-\infty }^{+\infty }d\xi \;\hat{N}{\left(\xi \right)}^{†}\hat{N}\left(\xi \right)=1\;,$$

where the right-hand side is treated as the identity operator. For the pre-measurement state $\hat{\rho }$, the measurement leads to the probability density function

(16) $$W_{\hat{\rho }}\left(\xi \right)=\mathrm{Tr}\left(\hat{N}{\left(\xi \right)}^{†}\hat{N}\left(\xi \right)\hat{\rho }\right)=\int_{-\infty }^{+\infty }{\left|g\left(\xi -x\right)\right|}^{2}\;w_{\hat{\rho }}\left(x\right)\;dx\;.$$

This should be used instead of $w_{\hat{\rho }}\left(x\right)=\langle x|\hat{\rho }|x\rangle$. When the acceptance function is sufficiently narrow, we will obtain a good “footprint” of $w_{\hat{\rho }}\left(x\right)$. Let ζ↦f(ζ) be another acceptance function that also obeys the normalization condition. A finite-resolution measurement of the legitimate wavenumber is described by some set N of operators

(17) $$\hat{M}\left(\zeta \right):=\int_{-\infty }^{+\infty }dk\;f\left(\zeta -k\right)\;|k\rangle \langle k|\;.$$

Here, the initial resolution $K=\left\{|k\rangle \langle k|\right\}$ is replaced with $M=\left\{\hat{M}\left(\zeta \right)\right\}$. Instead of $u_{\hat{\rho }}\left(k\right)=\langle k|\hat{\rho }|k\rangle$, we actually deal with the probability density function

(18) $$U_{\hat{\rho }}\left(\zeta \right)=\mathrm{Tr}\left(\hat{M}{\left(\zeta \right)}^{†}\hat{M}\left(\zeta \right)\hat{\rho }\right)=\int_{-\infty }^{+\infty }{\left|f\left(\zeta -k\right)\right|}^{2}\;u_{\hat{\rho }}\left(k\right)\;dk\;,$$

For good acceptance functions, a distortion of statistics will be small. The Gaussian distribution is a typical form of such functions [15]. We will assume that a behavior of acceptance functions is qualitatively similar.

## 3. On Successive Measurements of Observables in General

In this section, we generally formulate the question with respect to two successive measurements of observables with continuous spectra. It is more sophisticated than an intuitive obvious treatment of successive measurements on a finite-dimensional system. The latter allows us to deal with projective measurements, since all observables have a discrete spectrum. Such an approach is not meaningful for the case of position and momentum. On the other hand, the finite-dimensional case is important for understanding basic formulations related to continuous observables. To motivate our approach, we briefly review entropic uncertainty relations for successive projective measurements. Further, we will present a suitable reformulation for the case of position and momentum. Together with the entropic formulation, other approaches to express uncertainties in quantum measurements are of interest. In particular, modern investigations are based on the sum of variances [63,64], majorization relations [65,66,67,68,69], and the method of effective anticommutators [70]. The authors of [71] discussed some surprising results that may occur in application of entropic measures to quantify uncertainties in quantum measurements. These questions are beyond the scope of our consideration.

Scenarios with successive measurements are of interest for several reasons. The concept of wave function reduction assumes that we perform at least two successive measurements on a system (see for example Section 5.5 of [72]). By ${\hat{\Lambda }}_{a}\in A$, we denote a projector onto the *a*-th eigenspace of finite-dimensional observable $\hat{A}$. For the pre-measurement state $\hat{\rho }$, the probability of outcome *a* is written as $\mathrm{Tr}({\hat{\Lambda }}_{a}\hat{\rho })$. Such probabilities form a discrete distribution, from which we calculate quantities of interest. By $R_{\alpha }\left(A;\hat{\rho }\right)$ and $H_{\alpha }\left(A;\hat{\rho }\right)$, we further mean the entropies (1) and (3) calculated with the probabilities $\mathrm{Tr}({\hat{\Lambda }}_{a}\hat{\rho })$. After the measurement of $\hat{A}$, we measure another observable $\hat{B}$. It is actually described by the set $B=\{{\hat{\Pi }}_{b}\}$. Note that subsequent measurements are assumed to be performed with a new ensemble of states. The latter differs from traditional uncertainty relations in the preparation scenario. Scenarios with successive measurement are fixed by the used form of post-first-measurement states [16].

In the first scenario, the second measurement is performed on the state immediately following the first measurement with completely erased information. Here, the pre-measurement state of the second measurement is expressed as [14]

(19) $$\Upsilon_{A}\left(\hat{\rho }\right)=\sum_{a}{\hat{\Lambda }}_{a}\hat{\rho }{\hat{\Lambda }}_{a}\;.$$

To characterize the amount of uncertainty in two successive measurements, we will use quantities of the form

(20) $$R_{\alpha }\left(A;\hat{\rho }\right)+R_{\gamma }(B;\Upsilon_{A}\left(\hat{\rho }\right))\;,$$

and similarly with the corresponding Tsallis entropies. In the second scenario of successive measurements, we assume that the result of the first measurement is maintained. A focus on actual measurement outcomes is typical for the so-called selective measurements. For example, incoherent selective measurements are used in the formulation of monotonicity of coherence measures [73]. Coherence quantifiers can be defined with entropic functions of the Tsallis [74] and Rényi types [75]. In effect, the second measurement will be performed on the post-first-measurement state selected with respect to the actual outcome [16,17]. Due to the Lüders reduction rule [76], this state is written as

(21) $${\hat{\tau }}_{a}={\left(\mathrm{Tr}({\hat{\Lambda }}_{a}\hat{\rho })\right)}^{-1}\;{\hat{\Lambda }}_{a}\hat{\rho }{\hat{\Lambda }}_{a}\;,$$

whenever $\mathrm{Tr}({\hat{\Lambda }}_{a}\hat{\rho })\neq 0$. Measuring the observable $\hat{B}$ in each ${\hat{\tau }}_{a}$, we obtain the corresponding entropy $R_{\gamma }\left(B;{\hat{\tau }}_{a}\right)$. Averaging over all *a*, we introduce the quantity

(22) $$\sum_{a}\mathrm{Tr}\left({\hat{\Lambda }}_{a}\hat{\rho }\right)\;R_{\gamma }\left(B;{\hat{\tau }}_{a}\right)=\sum_{a}\mathrm{Tr}\left({\hat{\Lambda }}_{a}\hat{\rho }\right)\;R_{\alpha }\left(A;{\hat{\tau }}_{a}\right)+\sum_{a}\mathrm{Tr}\left({\hat{\Lambda }}_{a}\hat{\rho }\right)\;R_{\gamma }\left(B;{\hat{\tau }}_{a}\right)\;.$$

Of course, the first sum in the right-hand side of (22) vanishes. Measuring $\hat{A}$ in its eigenstate leads to a deterministic probability distribution, whence $R_{\alpha }\left(A;{\hat{\tau }}_{a}\right)=0$ for all *a*. It is for this reason that only the left-hand side of (22) is used in studies of uncertainties in successive measurements of finite-dimensional observables. In a similar manner, we can rewrite (20) and (22) with the use of Tsallis’ entropies. For α=γ=1, the quantity (22) becomes the Shannon entropy averaged over all *a*. The authors of [16] utilized the latter as a measure of uncertainties in successive measurements. Uncertainty relations for successive projective measurements in terms of Rényi’s entropies were analyzed in [17]. Formally, the sums involved in (22) are similar to one of several existing definitions of conditional Rényi’s entropy. In more detail, these definitions are discussed [77]. The simplest of them just leads to expressions of the form (22). Moreover, the two kinds of conditional Tsallis entropy are known in the literature [78,79]. More properties of generalized conditional entropies are discussed in [80].

Let us proceed to exact formulations for successive measurements of position and momentum. One cannot provide states in which the measurement of position or momentum gives exactly one particular value. Instead, we deal with well localized states of finite or even small scales. Following [48], the right-hand side of (22) will be used in extending the second scenario to the position-momentum case in the presence of a minimal length. Suppose that the first applied measurement aims to measure momentum. The authors of [15] mentioned how the post-first-measurement state should be posed. In our notation, we write

(23) $$\Phi_{M}\left(\hat{\rho }\right)=\int_{-\infty }^{+\infty }d\zeta \;\hat{M}\left(\zeta \right)\hat{\rho }\hat{M}{\left(\zeta \right)}^{†}\;.$$

This expression replaces the formula (19) suitable for observables with a purely discrete spectrum. The following important fact should be pointed out. If we again measure momentum, but now with the state (23), then it will result in the same probability distribution function. It can be derived from (17) that

(24) $$\langle k|\hat{\rho }|k\rangle =\langle k|\Phi_{M}\left(\hat{\rho }\right)|k\rangle \;,\;U_{\hat{\rho }}\left(\zeta \right)=U_{\Phi_{M}\left(\hat{\rho }\right)}\left(\zeta \right)\;.$$

Such relations may be interpreted as a mild version of the repeatability concept. For strictly positive α≠1, the Rényi α-entropy $R_{\alpha }\left(M;\hat{\rho }\right)$ is given by substituting $U_{\hat{\rho }}\left(\zeta \right)$ into (5). The standard differential entropy $H_{1}\left(M;\hat{\rho }\right)$ can be obtained within the limit α→1. Also, the Rényi α-entropy $R_{\alpha }\left(p_{M}^{\left(\delta \right)};\hat{\rho }\right)$ is defined by (1) by substituting probabilities defined through a discretization of the ζ-axis. When the first measurement is described by the set N, the post-first-measurement state is specified as

(25) $$\Phi_{N}\left(\hat{\rho }\right)=\int_{-\infty }^{+\infty }d\xi \;\hat{N}\left(\xi \right)\hat{\rho }\hat{N}{\left(\xi \right)}^{†}\;.$$

Let $\hat{\rho }$ denote the state right before the sequence of successive measurements. In the first scenario of successive measurements, we will characterize uncertainties by entropic quantities of the form

(26) $$R_{\alpha }\left(M;\hat{\rho }\right)+R_{\gamma }\left(N;\Phi_{M}\left(\hat{\rho }\right)\right)\;,\;R_{\alpha }\left(M;\Phi_{N}\left(\hat{\rho }\right)\right)+R_{\gamma }\left(N;\hat{\rho }\right)\;.$$

The former of the two sums concerns the case in which momentum is measured. Another useful approach is to calculate entropies with binning. For instance, sampling of the function (18) into bins between marks $\zeta_{j}$ gives a discrete probability distribution $p_{M}^{\left(\delta \right)}$. In the second measurement, entropies can be taken with binning between some marks $\xi_{k}$. By $p_{N}^{\left(\delta \right)}$, we mean the corresponding probability distribution. This approach leads to the characteristic quantities

(27) $$R_{\alpha }\left(p_{M}^{\left(\delta \right)};\hat{\rho }\right)+R_{\gamma }\left(p_{N}^{\left(\delta \right)};\Phi_{M}\left(\hat{\rho }\right)\right)\;,\;R_{\alpha }\left(p_{M}^{\left(\delta \right)};\Phi_{N}\left(\hat{\rho }\right)\right)+R_{\gamma }\left(p_{N}^{\left(\delta \right)};\hat{\rho }\right)\;.$$

In a similar manner, we formulate entropic measures of the Tsallis type. As was already mentioned, such entropies will be taken only with binning.

The second scenario of successive measurements prescribes that each actual result of the first measurement should be retained. Assuming $U_{\hat{\rho }}\left(\zeta \right)\neq 0$ in the corresponding domain, we now consider the normalized output state

(28) $$\hat{\varrho }\left(\zeta \right)=U_{\hat{\rho }}{\left(\zeta \right)}^{-1}\hat{M}\left(\zeta \right)\hat{\rho }\hat{M}{\left(\zeta \right)}^{†}\;.$$

Each $\hat{\varrho }\left(\zeta \right)$ is used as one of possible pre-measurement states in the second measurement. Similarly to (22), we then consider the quantity

(29) $$\int_{-\infty }^{+\infty }R_{\alpha }\left(M;\hat{\varrho }\left(\zeta \right)\right)U_{\hat{\rho }}\left(\zeta \right)\;d\zeta +\int_{-\infty }^{+\infty }R_{\gamma }\left(N;\hat{\varrho }\left(\zeta \right)\right)U_{\hat{\rho }}\left(\zeta \right)\;d\zeta \;.$$

When position is measured first, particular outputs are of the form

(30) $$\hat{\sigma }\left(\xi \right)=W_{\hat{\rho }}{\left(\xi \right)}^{-1}\hat{N}\left(\xi \right)\hat{\rho }\hat{N}{\left(\xi \right)}^{†}\;.$$

To describe the amount of uncertainty here, we rewrite (29) with $\hat{\sigma }\left(\xi \right)$ instead of $\hat{\varrho }\left(\zeta \right)$ and $W_{\hat{\rho }}\left(\xi \right)$ instead of $U_{\hat{\rho }}\left(\zeta \right)$. We will also utilize entropic uncertainty relations with binning. Here, one replaces (29) with

(31) $$\int_{-\infty }^{+\infty }R_{\alpha }\left(p_{M}^{\left(\delta \right)};\hat{\varrho }\left(\zeta \right)\right)U_{\hat{\rho }}\left(\zeta \right)\;d\zeta +\int_{-\infty }^{+\infty }R_{\gamma }\left(p_{N}^{\left(\delta \right)};\hat{\varrho }\left(\zeta \right)\right)U_{\hat{\rho }}\left(\zeta \right)\;d\zeta \;,$$

and similarly with the Tsallis entropies. Quantities of the form (31) concern successive measurements, in which position is measured after momentum. When position is measured by the first, we rewrite such expressions with $\hat{\sigma }\left(\xi \right)$ and $W_{\hat{\rho }}\left(\xi \right)$. In the paper [48], the above treatment of successive measurements was considered for general canonically conjugate operators. This approach to the concept of canonical conjugacy is based on the Pegg–Barnett formalism [50]. The Pegg–Barnett formalism was originally proposed to explain a Hermitian phase operator [81,82]. Entropic uncertainty relations on the base of this formalism were examined in [51,83,84].

## 4. Main Results

In this section, we shall formulate entropic uncertainty relations for successive measurements within the GUP case. For this case, preparation uncertainty relations with a correction term were derived in [49]. For the convenience of further calculations, the prepared pre-measurement state will be denoted by $\hat{\omega }$. Due to [49], we have

(32) $$H_{1}\left(M;\hat{\omega }\right)+H_{1}\left(N;\hat{\omega }\right)\geq H_{1}\left(K;\hat{\omega }\right)+H_{1}\left(X;\hat{\omega }\right)\geq \ln\left(e\pi \right)+{\langle \ln(1+\beta {\hat{k}}^{2})\rangle }_{\hat{\omega }}\;.$$

The well-known bound ln(eπ) corresponds to the entropic uncertainty relation of Beckner [42] and Białynicki-Birula and Mycielski [43]. The second term in the right-hand side of (32) reflects the fact that the legitimate momentum of the commutation relation (6) is given by $\hbar \hat{k}$. Here, the wavenumber operator $\hat{q}$ plays an auxiliary role. Note that this correction term depends on the pre-measurement state. As some numerical results in [85] later showed, the presented correction is sufficiently tight. It is similar to the correction term obtained in the Robertson formulation (8). However, the inequality (32) is a preparation uncertainty relation.

Suppose now that we measure momentum by the first and position by the second. In the first scenario, the pre-measurement state $\hat{\rho }$ leads to the post-first-measurement state $\Phi_{M}\left(\hat{\rho }\right)$. Due to (24), we immediately write

(33) $$H_{1}\left(M;\hat{\rho }\right)=H_{1}\left(M;\Phi_{M}\left(\hat{\rho }\right)\right)\;,\;{\langle \ln(1+\beta {\hat{k}}^{2})\rangle }_{\hat{\rho }}={\langle \ln(1+\beta {\hat{k}}^{2})\rangle }_{\Phi_{M}\left(\hat{\rho }\right)}\;.$$

Substituting $\hat{\omega }=\Phi_{M}\left(\hat{\rho }\right)$ into (32) and using (33), we easily get

(34) $$H_{1}\left(M;\hat{\rho }\right)+H_{1}\left(N;\Phi_{M}\left(\hat{\rho }\right)\right)\geq \ln\left(e\pi \right)+{\langle \ln(1+\beta {\hat{k}}^{2})\rangle }_{\hat{\rho }}\;.$$

This is an entropic uncertainty relation in the first scenario of successive measurements such that momentum is measured by the first. The corresponding lower bound is the same as in the preparation scenario. It is not the case, when we measure position by the first and momentum by the second. Putting $\hat{\omega }=\Phi_{N}\left(\hat{\rho }\right)$ into (32) finally gives

(35) $$H_{1}\left(N;\hat{\rho }\right)+H_{1}\left(M;\Phi_{N}\left(\hat{\rho }\right)\right)\geq \ln\left(e\pi \right)+{\langle \ln(1+\beta {\hat{k}}^{2})\rangle }_{\Phi_{N}\left(\hat{\rho }\right)}\;.$$

The correction term in the right-hand side of (35) is similar in form but should be calculated with the post-first-measurement state $\Phi_{N}\left(\hat{\rho }\right)$. Taking β=0, the above entropic bounds for successive measurements do not differ from the bound in the preparation scenario. Here, we see a manifestation of the deformed commutation relation (6). The latter disturbs a certain symmetry between position and momentum.

Let us proceed to the second scenario of successive measurements. Suppose again that momentum is measured by the first. Substituting $\hat{\omega }=\hat{\varrho }\left(\zeta \right)$ into (32), we multiply it by $U_{\hat{\rho }}\left(\zeta \right)$ and then integrate with respect to ζ. This results in the inequality

(36) $$\begin{array}{c} \int_{-\infty }^{+\infty }H_{1}\left(M;\hat{\varrho }\left(\zeta \right)\right)U_{\hat{\rho }}\left(\zeta \right)\;d\zeta +\int_{-\infty }^{+\infty }H_{1}\left(N;\hat{\varrho }\left(\zeta \right)\right)U_{\hat{\rho }}\left(\zeta \right)\;d\zeta \\ \geq \ln\left(e\pi \right)+\int_{-\infty }^{+\infty }{\langle \ln(1+\beta {\hat{k}}^{2})\rangle }_{\hat{\varrho }\left(\zeta \right)}\;U_{\hat{\rho }}\left(\zeta \right)\;d\zeta \;. \end{array}$$

Using (28), the second term in the right-hand side of (36) can be simplified, viz.,

(37) $$\begin{array}{c} \int_{-\infty }^{+\infty }d\zeta \;U_{\hat{\rho }}\left(\zeta \right)\int_{-\infty }^{+\infty }dk\;\ln\left(1+\beta k^{2}\right)\;\langle k|\hat{\varrho }\left(\zeta \right)|k\rangle \\ =\int_{-\infty }^{+\infty }d\zeta \;\int_{-\infty }^{+\infty }dk\;\ln\left(1+\beta k^{2}\right)\;\langle k|\hat{M}\left(\zeta \right)\hat{\rho }\hat{M}{\left(\zeta \right)}^{†}|k\rangle \\ =\int_{-\infty }^{+\infty }dk\;\ln\left(1+\beta k^{2}\right)\;\langle k|\hat{\rho }|k\rangle \int_{-\infty }^{+\infty }d\zeta \;{\left|f\left(\zeta -k\right)\right|}^{2}\;. \end{array}$$

In the right-hand side of (37), the last integral with respect to ζ is equal to 1. For the second scenario of successive measurements, we obtain

(38) $$\int_{-\infty }^{+\infty }H_{1}\left(M;\hat{\varrho }\left(\zeta \right)\right)U_{\hat{\rho }}\left(\zeta \right)\;d\zeta +\int_{-\infty }^{+\infty }H_{1}\left(N;\hat{\varrho }\left(\zeta \right)\right)U_{\hat{\rho }}\left(\zeta \right)\;d\zeta \geq \ln\left(e\pi \right)+{\langle \ln(1+\beta {\hat{k}}^{2})\rangle }_{\hat{\rho }}\;.$$

Hence, entropic uncertainty relations (34) and (38) are obtained with the same lower bound calculated with the pre-measurement state. Let us consider the case when position is measured by the first. Substituting $\hat{\omega }=\hat{\sigma }\left(\zeta \right)$ into (32), we multiply it by $W_{\hat{\rho }}\left(\xi \right)$ and integrate with respect to ξ, whence

(39) $$\begin{array}{c} \int_{-\infty }^{+\infty }H_{1}\left(M;\hat{\sigma }\left(\xi \right)\right)W_{\hat{\rho }}\left(\xi \right)\;d\xi +\int_{-\infty }^{+\infty }H_{1}\left(N;\hat{\sigma }\left(\xi \right)\right)W_{\hat{\rho }}\left(\xi \right)\;d\xi \\ \geq \ln\left(e\pi \right)+\int_{-\infty }^{+\infty }{\langle \ln(1+\beta {\hat{k}}^{2})\rangle }_{\hat{\sigma }\left(\xi \right)}\;W_{\hat{\rho }}\left(\xi \right)\;d\xi \;. \end{array}$$

In the right-hand side of (39), the second integral is a correction term averaged over particular outputs $\hat{\sigma }\left(\xi \right)$. In general, an expression for this term cannot be simplified without additional assumptions. We have already seen how the relation (35) differs from (34). The formula (39) differs from (38) in a similar vein. In the presence of a minimal length, the preparation uncertainty relation (32) remains valid for successive measurements, when momentum is measured by the first. Otherwise, it should be reformulated.

Entropic uncertainty relations with binning can be treated in a similar manner. Using some discretization of axes, we take into account sufficiently typical setup. This approach also leads to entropic functions with only positive values. In contrast, differential entropies can generally have arbitrary signs. In the case of momentum measurements, values $\zeta_{i}$ denote the ends of intervals $\delta \zeta_{i}=\zeta_{i+1}-\zeta_{i}$. For the prepared state $\hat{\omega }$, we deal with probabilities

(40) $$p_{i}^{\left(\delta \right)}:=\int_{\zeta_{i}}^{\zeta_{i+1}}U_{\hat{\omega }}\left(\zeta \right)\;d\zeta \;,$$

which form the discrete distribution $p_{M}^{\left(\delta \right)}$. Using (40), one calculates the Shannon entropy $H_{1}\left(p_{M}^{\left(\delta \right)};\hat{\omega }\right)$. In a similar way, we discretize the ξ-axes into bins $\delta \xi_{j}=\xi_{j+1}-\xi_{j}$ with the resulting distribution $p_{N}^{\left(\delta \right)}$. It can be shown that

(41) $$H_{1}\left(p_{M}^{\left(\delta \right)};\hat{\omega }\right)+H_{1}\left(p_{N}^{\left(\delta \right)};\hat{\omega }\right)\geq \ln\;\left(\frac{e\pi }{\delta \zeta \;\delta \xi }\right)+{\langle \ln(1+\beta {\hat{k}}^{2})\rangle }_{\hat{\omega }}\;,$$

where $\delta \zeta =\max\delta \zeta_{i}$ and $\delta \xi =\max\delta \xi_{j}$. The formula (41) gives a preparation uncertainty relation with binning. It involves the same correction term due to the existence of a minimal length. To pose entropic uncertainty relations in the first scenario of successive measurements, we again use reasons that have lead to (34) and (35). Finally, one gets

(42) $$\begin{array}{cc} H_{1}\left(p_{M}^{\left(\delta \right)};\hat{\rho }\right)+H_{1}\left(p_{N}^{\left(\delta \right)};\Phi_{M}\left(\hat{\rho }\right)\right) & \geq \ln\;\left(\frac{e\pi }{\delta \zeta \;\delta \xi }\right)+{\langle \ln(1+\beta {\hat{k}}^{2})\rangle }_{\hat{\rho }}\;, \end{array}$$

(43) $$\begin{array}{cc} H_{1}\left(p_{N}^{\left(\delta \right)};\hat{\rho }\right)+H_{1}\left(p_{M}^{\left(\delta \right)};\Phi_{N}\left(\hat{\rho }\right)\right) & \geq \ln\;\left(\frac{e\pi }{\delta \xi \;\delta \zeta }\right)+{\langle \ln(1+\beta {\hat{k}}^{2})\rangle }_{\Phi_{N}\left(\hat{\rho }\right)}\;. \end{array}$$

In the second scenario of successive measurements, entropic uncertainty relations with binning are obtained in the form

(44) $$\begin{array}{cc} \int_{-\infty }^{+\infty }H_{1}\left(p_{M}^{\left(\delta \right)};\hat{\varrho }\left(\zeta \right)\right)U_{\hat{\rho }}\left(\zeta \right)\;d\zeta +\int_{-\infty }^{+\infty }H_{1}\left(p_{N}^{\left(\delta \right)};\hat{\varrho }\left(\zeta \right)\right)U_{\hat{\rho }}\left(\zeta \right)\;d\zeta & \geq \ln\;\left(\frac{e\pi }{\delta \zeta \;\delta \xi }\right)+{\langle \ln(1+\beta {\hat{k}}^{2})\rangle }_{\hat{\rho }}\;, \end{array}$$

(45) $$\begin{array}{cc} \int_{-\infty }^{+\infty }H_{1}\left(p_{N}^{\left(\delta \right)};\hat{\sigma }\left(\xi \right)\right)W_{\hat{\rho }}\left(\xi \right)\;d\xi +\int_{-\infty }^{+\infty }H_{1}\left(p_{M}^{\left(\delta \right)};\hat{\sigma }\left(\xi \right)\right)W_{\hat{\rho }}\left(\xi \right)\;d\xi & \geq \ln\;\left(\frac{e\pi }{\delta \xi \;\delta \zeta }\right)\\ & +\int_{-\infty }^{+\infty }{\langle \ln(1+\beta {\hat{k}}^{2})\rangle }_{\hat{\sigma }\left(\xi \right)}W_{\hat{\rho }}\left(\xi \right)\;d\xi \;. \end{array}$$

In the presence of a minimal length, distinctions of (43) and (45) from the corresponding preparation relations are concentrated in correction terms. In effect, these terms are not state-independent. On the other hand, entropic bounds of preparation uncertainty relations remain valid when momentum is measured by the first. The author of [49] also reported on state-independent entropic uncertainty relations in the presence of a minimal length. Such relations were posed in terms of the Rényi and Tsallis entropies with binning. An alteration of statistics due to a finite resolution of the measurements is also taken into account. When acceptance functions of measurement apparatuses are sufficiently spread, they lead to an increase of entropic lower bounds. To pose uncertainty relations formally, we introduce the following quantity [49],

(46) $$S_{f}:=\underset{\zeta }{\sup}\int_{-\infty }^{+\infty }\frac{{\left|f\left(\zeta -k\right)\right|}^{2}}{1+\beta k^{2}}\;dk\;,$$

where the acceptance function ζ↦f(ζ) corresponds to momentum measurements. Let $\hat{\omega }$ represent the prepared state. As was shown in [49], the existence of a minimal length leads to preparation uncertainty relations of the form

(47) $$R_{\alpha }\left(M;\hat{\omega }\right)+R_{\gamma }\left(N;\hat{\omega }\right)\geq \ln\;\left(\frac{\varkappa \pi }{S_{f}}\right).$$

Here, positive entropic parameters obey 1/α+1/γ=2 and

(48) $$\varkappa^{2}=\alpha^{1/(\alpha -1)}\gamma^{1/(\gamma -1)}\;.$$

In the limit α→1, the parameter ϰ becomes equal to *e*. When β=0, we clearly have $S_{f}=1$, so that the right-hand side of (47) reduces to ln(ϰπ). The latter is a known entropic bound for the case of usual position and momentum. For β>0 and physically reasonable acceptance functions, we obtain an improved lower due to $S_{f}<1$. It is important that the quantity (46) depends only on β and the actual acceptance function in momentum measurements. Preparation entropic uncertainty relations with binning are posed as follows [49]. Let probability density functions $U_{\hat{\omega }}\left(\zeta \right)$ and $W_{\hat{\omega }}\left(\xi \right)$ be sampled into discrete probability distributions. Then the corresponding Rényi and Tsallis entropies satisfy

(49) $$\begin{array}{cc} R_{\alpha }\left(p_{M}^{\left(\delta \right)};\hat{\omega }\right)+R_{\gamma }\left(p_{N}^{\left(\delta \right)};\hat{\omega }\right) & \geq \ln\;\left(\frac{\varkappa \pi }{S_{f}\;\delta \zeta \;\delta \xi }\right), \end{array}$$

(50) $$\begin{array}{cc} H_{\alpha }\left(p_{M}^{\left(\delta \right)};\hat{\omega }\right)+H_{\gamma }\left(p_{N}^{\left(\delta \right)};\hat{\omega }\right) & \geq \ln_{\nu }\;\left(\frac{\varkappa \pi }{S_{f}\;\delta \zeta \;\delta \xi }\right), \end{array}$$

where 1/α+1/γ=2 and ν=max{α,γ}.

Due to equalities of the form (24), the preparation uncertainty relations (47), (49), and (50) are immediately converted into relations for successive measurements. In the first scenario, we obtain

(51) $$R_{\alpha }\left(M;\hat{\rho }\right)+R_{\gamma }\left(N;\Phi_{M}\left(\hat{\rho }\right)\right)\geq \ln\;\left(\frac{\varkappa \pi }{S_{f}}\right),$$

where 1/α+1/γ=2 and the momentum measurement is assumed to be made by the first. When position is measured by the first, we replace $\hat{\rho }$ with $\Phi_{N}\left(\hat{\rho }\right)$ and $\Phi_{M}\left(\hat{\rho }\right)$ with $\hat{\rho }$ in the left-hand side of (51). For 1/α+1/γ=2 and ν=max{α,γ}, entropic uncertainty relations with binning are written as

(52) $$\begin{array}{cc} R_{\alpha }\left(p_{M}^{\left(\delta \right)};\hat{\rho }\right)+R_{\gamma }\left(p_{N}^{\left(\delta \right)};\Phi_{M}\left(\hat{\rho }\right)\right) & \geq \ln\;\left(\frac{\varkappa \pi }{S_{f}\;\delta \zeta \;\delta \xi }\right), \end{array}$$

(53) $$\begin{array}{cc} H_{\alpha }\left(p_{M}^{\left(\delta \right)};\hat{\rho }\right)+H_{\gamma }\left(p_{N}^{\left(\delta \right)};\Phi_{M}\left(\hat{\rho }\right)\right) & \geq \ln_{\nu }\;\left(\frac{\varkappa \pi }{S_{f}\;\delta \zeta \;\delta \xi }\right). \end{array}$$

The same entropic lower bounds hold, when position is measured by the first. We refrain from presenting the details here. In the second scenario of successive measurements, one immediately gets

(54) $$\int_{-\infty }^{+\infty }R_{\alpha }\left(M;\hat{\varrho }\left(\zeta \right)\right)U_{\hat{\rho }}\left(\zeta \right)\;d\zeta +\int_{-\infty }^{+\infty }R_{\gamma }\left(N;\hat{\varrho }\left(\zeta \right)\right)U_{\hat{\rho }}\left(\zeta \right)\;d\zeta \geq \ln\;\left(\frac{\varkappa \pi }{S_{f}}\right),$$

where 1/α+1/γ=2 and the momentum measurement is assumed to be made by the first. Replacing $\hat{\varrho }\left(\zeta \right)$ with $\hat{\sigma }\left(\xi \right)$ and $U_{\hat{\rho }}\left(\zeta \right)$ with $W_{\hat{\rho }}\left(\xi \right)$, we resolve the case when position is measured by the first. For 1/α+1/γ=2 and ν=max{α,γ}, entropic uncertainty relations with binning are expressed as

(55) $$\begin{array}{cc} \int_{-\infty }^{+\infty }R_{\alpha }\left(p_{M}^{\left(\delta \right)};\hat{\varrho }\left(\zeta \right)\right)U_{\hat{\rho }}\left(\zeta \right)\;d\zeta +\int_{-\infty }^{+\infty }R_{\gamma }\left(p_{N}^{\left(\delta \right)};\hat{\varrho }\left(\zeta \right)\right)U_{\hat{\rho }}\left(\zeta \right)\;d\zeta & \geq \ln\;\left(\frac{\varkappa \pi }{S_{f}\;\delta \zeta \;\delta \xi }\right), \end{array}$$

(56) $$\begin{array}{cc} \int_{-\infty }^{+\infty }H_{\alpha }\left(p_{M}^{\left(\delta \right)};\hat{\varrho }\left(\zeta \right)\right)U_{\hat{\rho }}\left(\zeta \right)\;d\zeta +\int_{-\infty }^{+\infty }H_{\gamma }\left(p_{N}^{\left(\delta \right)};\hat{\varrho }\left(\zeta \right)\right)U_{\hat{\rho }}\left(\zeta \right)\;d\zeta & \geq \ln_{\nu }\;\left(\frac{\varkappa \pi }{S_{f}\;\delta \zeta \;\delta \xi }\right). \end{array}$$

When position is measured first, we merely replace here $\hat{\varrho }\left(\zeta \right)$ with $\hat{\sigma }\left(\xi \right)$ and $U_{\hat{\rho }}\left(\zeta \right)$ with $W_{\hat{\rho }}\left(\xi \right)$. Thus, state-independent entropic lower bounds of preparation uncertainty relation remain valid for scenarios with successive measurements. The existence of a minimal length is taken into account due to the quantity (46). In the case α=γ=1, the above relations are expressed via the Shannon entropies. We have also obtained state-dependent entropic uncertainty relations such as (35), (39), (43), and (45). Their formulations differ from preparation uncertainty relations since they depend on the quantum state immediately following the first measurement.

## 5. Conclusions

We have formulated entropic uncertainty relations for successive measurements in the presence of a minimal length. The presented formulation is explicitly dependent on the order of the measurements, though the bounds themselves may not be optimal. The problem of a minimal observable length is related to efforts to describe gravitation at the quantum level. In effect, the generalized uncertainty principle restricts the space of acceptable wave packets. Scenarios with successive measurements are interesting for several reasons. The traditional scenario of preparation uncertainty relations is insufficient even from the viewpoint of Heisenberg’s thought experiment [1]. Successive measurements of position and momentum cannot be treated as projective even within ordinary quantum mechanics. The GUP case implies additional limitation for a spatial width of the acceptance function in position measurements. Thus, entropic measures of uncertainty should be formulated differently from the finite-dimensional case. One of distinctions concerns a proper form of the state immediately following the first measurement. The post-first-measurement state was chosen according to the two possible scenarios. Uncertainty relations in terms of Shannon entropies contain a state-dependent correction term. Hence, entropic lower bounds for successive measurements generally differ from lower bounds involved into preparation uncertainty relations. We also formulated state-independent uncertainty bounds in terms of Rényi entropies and, with binning, in terms of Tsallis entropies. In the presence of a minimal length, state-independent entropic lower bounds of preparation uncertainty relations remain valid for scenarios with successive measurements. When acceptance functions of measurement apparatuses are sufficiently well spread, the existing entropic lower bounds are improved.
